# Sepsis-Associated Brain Dysfunction: A Review of Current Literature

**DOI:** 10.3390/ijerph17165852

**Published:** 2020-08-12

**Authors:** Piotr F. Czempik, Michał P. Pluta, Łukasz J. Krzych

**Affiliations:** 1 Department of Anaesthesiology and Intensive Care, Faculty of Medical Sciences in Katowice, Medical University of Silesia, Katowice, Medyków 14, 40-752 Katowice, Poland; lkrzych@sum.edu.pl; 2 St. Barbara’s Memorial Hospital No. 5 Trauma Center, Plac Medyków 1, 41-200 Sosnowiec, Poland; michal_p2@o2.pl; 3 Students’ Scientific Society, Department of Anaesthesiology and Intensive Care, Faculty of Medical Sciences in Katowice, Medical University of Silesia, Katowice, Medyków 14, 40-752 Katowice, Poland;

**Keywords:** diagnosis, epidemiology, management, pathophysiology, prevention, sepsis-associated brain dysfunction, sepsis-associated encephalopathy

## Abstract

Sepsis-associated brain dysfunction (SABD) may be the most common type of encephalopathy in critically ill patients. SABD develops in up to 70% of septic patients and represents the most frequent organ insufficiency associated with sepsis. It presents with a plethora of acute neurological features and may have several serious long-term psychiatric consequences. SABD might cause various pathological changes in the brain through numerous mechanisms. Clinical neurological examination is the basic screening method for SABD, although it may be challenging in subjects receiving with opioids and sedative agents. As electrographic seizures and periodic discharges might be present in 20% of septic patients, screening with electroencephalography (EEG) might be useful. Several imaging techniques have been suggested for non-invasive assessment of structure and function of the brain in SABD patients; however, their usefulness is rather limited. Although several experimental therapies have been postulated, at the moment, no specific treatment exists. Clinicians should focus on preventive measures and optimal management of sepsis. This review discusses epidemiology, clinical presentation, pathology, pathophysiology, diagnosis, management, and prevention of SABD.

## 1. Introduction

Although significant progress has recently been made in increasing awareness among healthcare providers and in recognition and management [1], sepsis and septic shock remain a major healthcare problem worldwide. An integral component of sepsis is an acute organ dysfunction of multiple organs and organ systems. According to the most recent consensus, sepsis is defined as life-threatening organ dysfunction caused by a dysregulated host response to infection [2]. As intensive care units (ICUs) are well placed for managing organ failures, sepsis is a frequent ICU admission diagnosis [3]. One of the organs affected in sepsis is the brain, and sepsis-associated brain dysfunction (SABD) is probably the most common type of encephalopathy in the ICU. SABD is defined as diffuse brain dysfunction caused by infection outside the central nervous system (CNS) and is a diagnosis of exclusion. There should be no other diagnoses explaining the neurological status of a patient (e.g., CNS infection). SABD might develop in up to 70% of septic patients [4], especially in patients with confirmed bacteremia [5], and represents the most frequent organ insufficiency associated with sepsis [6]. Disturbed consciousness in sepsis significantly increases mortality. The prevalence and dismal prognosis of patients presenting with SABD only highlights the imperative to better understand the condition, improve its diagnosis and treatment, and introduce preventive measures. In this review, based on the latest research, we discuss the following aspects of SABD: epidemiology, clinical presentation, pathogenesis/pathophysiology, diagnosis, prevention, and management.

## 2. Materials and Methods

### Epidemiology

The prevalence of SABD is difficult to assess due to its various neurological manifestations and the fact that many other factors, apart from sepsis, may lead to brain dysfunction in septic patients. Additionally, sedation makes assessment of SABD difficult. Young et al. reported that among 69 patients with fever and positive microbial cultures, 32 had marked brain dysfunction, 17 showed mild encephalopathy, and 20 were neurologically asymptomatic [7]. A retrospective study evaluating ICU hospitalizations in a 3-year period reported sepsis-associated encephalopathy (SAE) in 17.7% of patients; however, this study was based on an old definition of sepsis (2011), lacked precise diagnostic criteria for SABD, and patients receiving sedative agents were excluded [8]. Even more than 50% of septic patients may show signs of SABD [9], even before admission to hospital [4]. SABD may constitute the most common type of encephalopathy in a heterogeneous ICU population. Up to 70% of septic patients may develop SABD. Deteriorating neurological status (as per the Glasgow Coma Scale (GCS)) in SABD leads to increasing mortality; hence, regular screening for and monitoring of brain dysfunction is crucial [8]. The prevalence of SABD depends also on the site and etiology of infection. Biliary and intestine infections and pathogens such as *Staphylococcus aureus*, *Enterococcus faecium*, *Acinetobacter* spp., *Pseudomonas aeruginosa*, and *Stenotrophomonas maltophilia*, are associated with a higher risk of SABD [8]. Due to the lack of definite diagnostic criteria for SABD and variable clinical manifestation, prevalence and mortality of SABP is difficult to assess.

## 3. Results

### Clinical Presentation

Due to use of opioids and sedative agents, accurate neurological assessment of patients hospitalized in the ICU might be impossible. There is a plethora of acute neurological features of SABD as well as long-term psychiatric consequences [7]. The long-term consequences of SABD might be present in up to 62% of patients [7]. Experimental research suggests that neuronal loss and reduced cholinergic signaling is responsible for the long-term consequences of SABD [8]. Table 1 lists various neurological features of SABD.

## 4. Discussion

### 4.1. Pathogenesis/ Pathophysiology

The pathologic changes detected in SABD include cerebral ischemia, hemorrhages [19], disseminated micro-abscesses [19,20,21,22], central pontine myelinolysis, multifocal necrotizing leukoencephalopathy [21], perivascular edema, swelling of astrocytic end-feet, and neuronal apoptosis [22]. These changes are present in the most severe cases.

The parts of the brain mostly involved in sepsis are the frontal cortex and the hippocampus, which leads to cognitive dysfunction [23]. There might be a brainstem dysfunction caused by the passage of inflammatory mediators through area postrema. Brainstem dysfunction may present as impaired alertness, impaired cardiovascular and immune control, and impaired brainstem reflexes [24]. Sepsis is associated with several neuroendocrine disorders: pituitary insufficiency, relative adrenal insufficiency, and impaired production of vasopressin [25]. Microglial activation affects the amygdala leading to psychological symptoms (anxiety, depression, post-traumatic stress disorder (PTSD)) [26].

**Figure 1:**
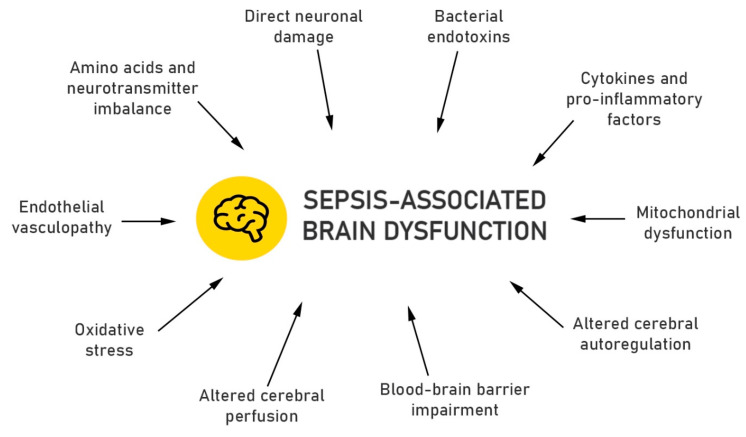
Proposed pathophysiologic changes in sepsis-associated brain dysfunction.

Potential pathophysiologic mechanisms leading to SABD are summarized in Figure 1.

Bacterial endotoxins (lipopolysaccharide, LPS) are bonded by circulating LPS-binding protein (LBP), which later forms a complex with a membrane-bound cluster of differentiation (CD) 14 receptors on monocytes, macrophages, and neutrophils, what induces synthesis of pro-inflammatory cytokines, such as tumor necrosis factor -α (TNF-α), interleukin - 1β (IL-1β), and interleukin-6 (IL-6) through Toll-like receptors (TLR) 2 and 4 [27,28].

Systemically produced cytokines (IL-1β, TNF-α, IL-6) activate microglia, causing the release of inflammatory mediators in the brain (e.g., transforming growth factor-beta1 and prostaglandin E2 cytokines), leading to disturbed neuronal function [27].

Pro-inflammatory cytokines in turn induce synthesis and secretion of nitric oxide (NO) and reactive oxygen radicals (ROS) [29]. Production of NO is initially increased; however, free radicals (such as superoxide anion) later react with NO forming peroxynitrite, which leads to a reduced NO bioavailability in the cerebrovascular bed. Experimental data using a fecal peritonitis Wistar rat model indicate that oxidative stress occurs early in sepsis and leads to decreased synthesis of adenotriphosphate (ATP) due to liver and skeletal muscle mitochondrial injury [30,31].

Oxidative stress leads to endothelial vasculopathy. Cytokine storm in SABD reduces concentrations of protein C and activated protein C and, along with endothelial vasculopathy, tips the balance of the hemostatic system toward coagulation and the formation of micro-thrombi, leading to tissue ischemia [32].

In normal conditions, cerebral vasodilation is mediated by nitric oxide, whereas cerebral vasoconstriction by endothelin [33]. In SABD, pro-inflammatory mediators (e.g., TNF-alpha, IFN-gamma, IL-1, IL-8) decrease NO, leading to increased cerebral arteriolar (40–200 μm) resistance with resultant decrease in cerebral blood flow and cerebral blood volume [34].

There may be an elevated level of NO leading to altered cerebral autoregulation. The coupling between blood flow and metabolism is lost. In patients with sepsis, there is a disturbed response of cerebral blood vessels to carbon dioxide [35].

Impairment of the blood–brain barrier (BBB) leads to a disturbed water transport, which is tightly regulated by aquaporin 4, resulting in perivascular edema, destruction of the astrocytic end-feet [19]. Through impaired BBB, leaked aromatic amino acids (AAAs) lead to an altered mental status [36]. Protein leaks through the impaired BBB and shows in cerebrospinal fluid (CSF) [37].

Concentrations of amino acids and neurotransmitters in the plasma and brain differ significantly between septic patients and healthy controls. AAAs are elevated in sepsis, whereas branched chain amino acids (BCAAs) are decreased [38]. BCAAs and AAAs compete for the same transporter in the BBB, so if BCAA levels are low, there will be an increase in the brain uptake of AAAs (SAE symptoms).

The concentration of glutamate, a major activating neurotransmitter in the brain, increases in sepsis. Glutamate works via activation of N-methyl-D-aspartate glutamate receptors, which in turn decreases cellular ATP. Lack of ATP leads to Na/K-ATPase inhibition and cellular edema.

The cholinergic stimulation through nicotinic and muscarinic receptors modulates memory, learning abilities, arousal level, and major cognitive functions. It is significantly reduced in SABD and may lead to disruption of the aforementioned brain functions. Anticholinergic medications are considered risk factors for delirium; however, cholinesterase inhibitors did not show efficacy when used in prevention or treatment [39].

Excessive concentrations of norepinephrine and dopamine have been associated with hyperactive delirium, making dopamine antagonists useful for acute symptom control. However, dopamine antagonists do not shorten the duration or decrease severity of delirium in critically ill patients [40].

### 4.2. Diagnosis

SABD is a diagnosis of exclusion. In order to diagnose SABD, factors other than sepsis have to be excluded: drug effects, electrolyte disturbances, metabolic disturbances, primary CNS pathology (meningitis, encephalitis, cerebral abscess, septic emboli), and conditions with non-infective systemic inflammatory response (e.g., burns, severe acute pancreatitis, trauma). Neurological examination is the basic screening method for SABD in septic patients. In mechanically ventilated patients, deeper levels of sedation are occasionally necessary and preclude clinical neurological assessment. In patients who are not sedated or lightly sedated, objective assessment can be used. The most common objective method is the Confusion Assessment Method for the ICU (CAM-ICU), which has been translated into several languages. Nevertheless, when applied by ICU nurses, CAM-ICU showed lower sensitivity than unstructured delirium assessments made by qualified nurses [41]. When compared to assessments made by expert teams of psychiatrists, geriatricians, and neurologists, CAM-ICU showed a sensitivity of 47% and specificity of 98% [42]. These two studies were performed specifically to diagnose delirium associated with sepsis.

When clinical assessment is difficult or not feasible, instrumental methods can be used. The most sensitive method is electroencephalography (EEG). Normal alpha waves (7.5–12.5 Hz) slow down and theta waves (4–8 Hz) emerge in patients with no, mild, and moderate clinical symptoms (confusion, delirium). The appearance of delta waves (4 Hz), generalization of triphasic waves, and more burst-suppression pattern are associated with severe symptoms (stupor, coma). Mortality increases with the malignancy of EEG wave forms [43]. Nevertheless, these abnormal EEG findings are not specific to SABD and are present in other types of encephalopathy [44]. A recent study showed an association between delirium in non-sedated septic patients and electrographic seizures. Electrographic seizures and periodic discharges were present in 20% of septic patients. These wave forms were independently associated with ICU mortality, confirming the link between SABD and mortality [45].

In mild cases, computed tomography (CT) and magnetic resonance imaging (MRI) examinations are usually normal. In more severe cases, non-specific structural changes can be detected (mostly MRI): leukoencephalopathy, cerebral infarction, cerebral atrophy, generalized lowered density in the white matter, vasogenic edema, changes in the corpus callosum, subcortical, and deep cerebellar regions [46,47,48]. The degree of detected imaging abnormalities is correlated with severity of sepsis. In ICU survivors, an association between lower brain volumes (MRI) and longer duration of delirium/worse cognitive impairment (up to a year) was found [49].

Transcranial Doppler may detect cerebral blood flow changes, enabling easy and non-invasive assessment [50]. A pulsatility index higher than 1.3 in the first 24 h may be indicative of brain dysfunction in sepsis (delirium) [51].

As another non-invasive method, measurement of optic nerve sheath diameter (ONSD) can detect elevated intracranial pressure in septic patients. Intracranial hypertension in septic patients might be a sign of SABD. Nevertheless, using ONSD for SABD screening requires further research [52].

In patients with sepsis and septic shock, biomarkers of glia (S100β) or neurons (neuron-specific enolase) were detected; however, they are not specific for SABD [53].

Elevated concentration of protein in CSF, a consequence of increased permeability of blood proteins through the BBB, might be indicative of SABD.

### 4.3. Management

Although several experimental therapies have been postulated [54,55,56], at the moment, there is no specific treatment for SABD. The optimal treatment of the underlying sepsis is crucial. In patients with severe sepsis and septic shock, decrease in cerebral perfusion pressure correlated with increased serum S100β levels [57]; therefore, attempts may be made to increase mean arterial pressure to mitigate brain injury.

A large multicenter randomized clinical trial carried out in American medical centers showed that a typical antipsychotic (haloperidol) or an atypical antipsychotic (ziprasidone) did not shorten the time patients were delirious or comatose, compared to a placebo. There were also no differences in mortality, ICU or hospital length stay [40].

An alpha-2 adrenergic agonist agent dexmedetomidine showed neuroprotective effects (inhibition of neuronal apoptosis, reduction in sepsis-associated inflammatory response, more delirium-free days) in septic patients compared to lorazepam [58]. The effect was probably due to inhibition of neuronal apoptosis and reduction of inflammatory response associated with sepsis. Sedation with dexmedetomidine, as compared to lorazepam, led to more encephalopathy-free days, shorted ventilation times, and lower mortality.

### 4.4. Prevention

As management of SABD is based on treatment of underlying sepsis, all patients at risk of sepsis should be screened. Through timely management of sepsis, it may be possible to prevent patients from developing SABD. Attention should be given to simple infections that may aggravate into sepsis.

The clinical symptoms of SABD may be detected prior to other symptoms of sepsis. According to Gofton et al., cognitive or mental changes associated with SABD can occur in susceptible patients up to 36–48 h before other systemic symptoms of sepsis. Such patients show unexplained reduction in level of consciousness but otherwise have normal neurological examination and neuroimaging [4]. Screening for delirium in order to identify patients prone to SABD is crucial here. In these patients, a search for infection should be started and appropriate therapy promptly introduced. The role of routine EEG in detection of SABD is not clear.

Elimination or reduction of the amount of sedation, especially benzodiazepines, showed a reduced incidence of sepsis-associated delirium; hence, introduction of minimal sedation policy should benefit ICU patients [59].

There are several modifiable factors associated with SABD (e.g., hypoglycemia, hyperglycemia, hypercapnia, hypernatremia) that should be prevented [60].

## 5. Conclusions

Sepsis-associated brain dysfunction is a multifactorial disorder with high prevalence and mortality among critically ill septic patients. SABD should we viewed as acute organ insufficiency in early phase and as a cause of numerous long-term psychiatric problems. Several imaging techniques have been suggested for non-invasive assessment of structure and function of the brain in SABD patients; however, their usefulness is rather limited. Although some potential therapies have been tested at the experimental level, there are no specific treatment options available so far. Clinicians should focus on preventive measures and optimal management of sepsis.

## Figures and Tables

**Table 1:** Clinical features of sepsis-associated brain dysfunction.

Acute Problems	Long-Term Problems
Fluctuations of Vigilance	Lower health-related quality of life [10,11,12,13,14]
Lethargy	Anxiety
Delirium	Post-traumatic stress disorder [15]/Post-sepsis syndrome [16]
Coma	Depression
Polyneuropathy	Suicidal behavior [17]
	Dementia [18]
